# Supplementary material for: The twist-and-squeeze activation of CARF-fused adenosine deaminase by cyclic oligoadenylates
Source: EMBO J. 2025 Oct 17;44(23):6919–43. doi: 10.1038/s44318-025-00578-y (PMC12669630; doi:10.1038/s44318-025-00578-y)
Supplement: Supplementary file 1 — Appendix [file 44318_2025_578_MOESM1_ESM.pdf]

Appendix for
The twist-and-squeeze activation of CARF-Fused Adenosine Deaminase by Cyclic Oligoadenylates

Table of Contents

Appendix Figure S1: Protein purification and functional characterizations of the CARF domain of *Taq*Cad1……2

Appendix Figure S2: Structural properties of *Taq*Cad1-cA_4…………………………….……………....................3

Appendix Figure S3: Maps of the plasmids used in the in vivo plasmid challenge assay......................................4

Appendix Figure S4: Structural comparison of *Taq*Cad1 with ADA...5

Appendix Figure S5: Structural comparison of *Taq*Cad1 with other Cad1 structures..6

Appendix Table S1: Statistics of cryo-EM data collection and processing …………………...………......................7

Appendix Table S2: Statistics of model refinement……………………………………………..…..................…......8

Appendix Table S3: Oligos used in this study.........……………………………………………………..............................9

Sequence Information………………………………………………………………………………..........................…...10

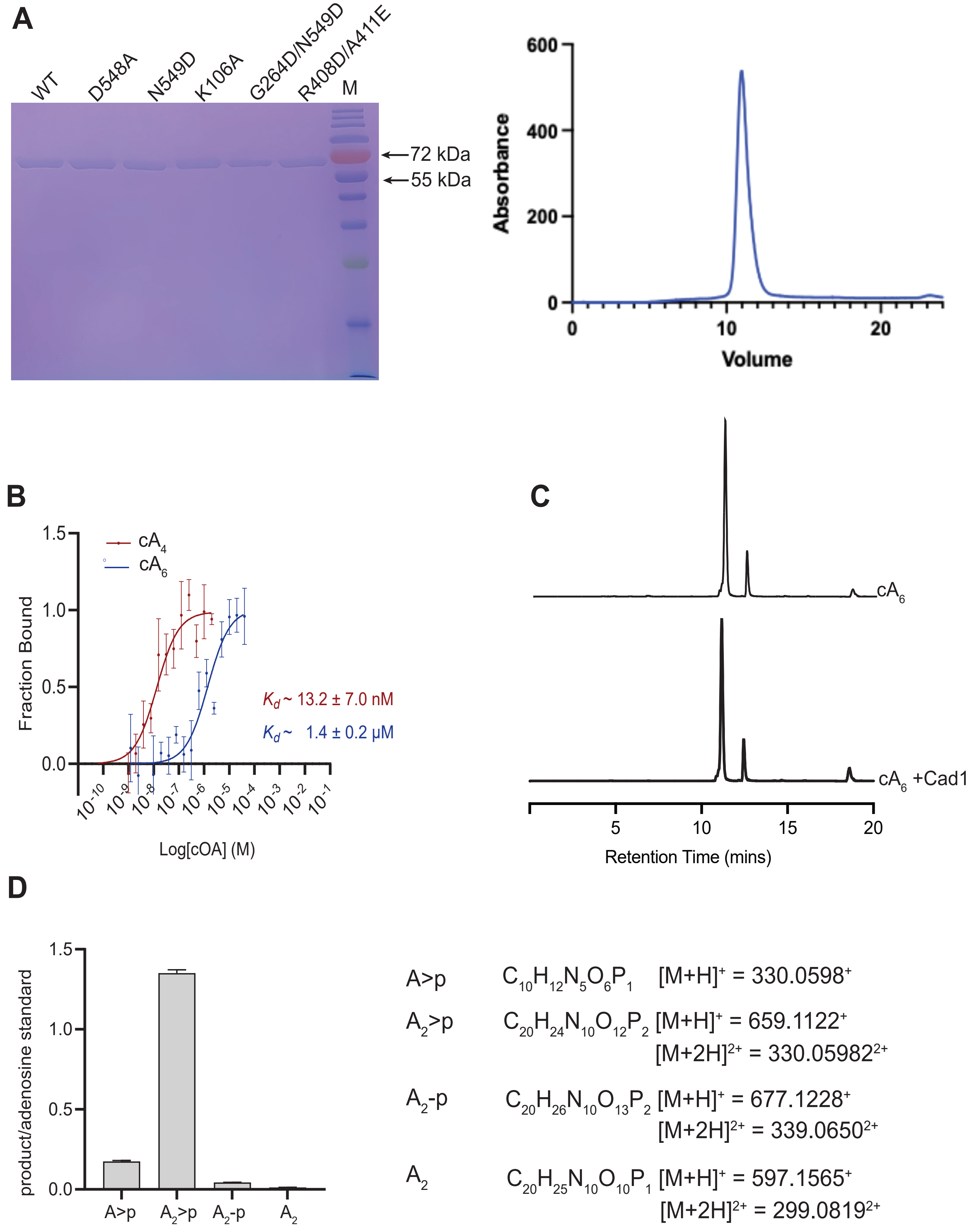

Appendix Figure S1. Protein purification and functional characterizations of the CARF domain of *Taq*Cad1.

(A) Left, protein purity of the wild-type (WT) and the mutants is assessed on an SDS-PAGE gel stained by Coomassie brilliant blue. Right, an example size exclusion chromatography elution profile of the wild-type protein monitored by 260 nm absorptance. **(B)** Microscale thermophoresis (MST) profiles of titrating *Taq*Cad1 with cA_4 (red) or cA_6 (black). The binding disassociation constant is estimated by fitting the binding isotherms to a one-site binding model. **(C)** High Performance Liquid Chromatography (HPLC) analysis of cA_6 molecules incubated with (lower) or without (upper) *Taq*Cad1. **(D)** Mass spectrometry analysis of the ring nuclease products of *Taq*Cad1 upon incubating with cA_4. The chemical formula and mass to charge ratios of each of the detected products are listed to the right. Error bars represent the standard deviation of the mean. Data represent the mean ± standard deviation of the mean (n = 3 independent experiments).

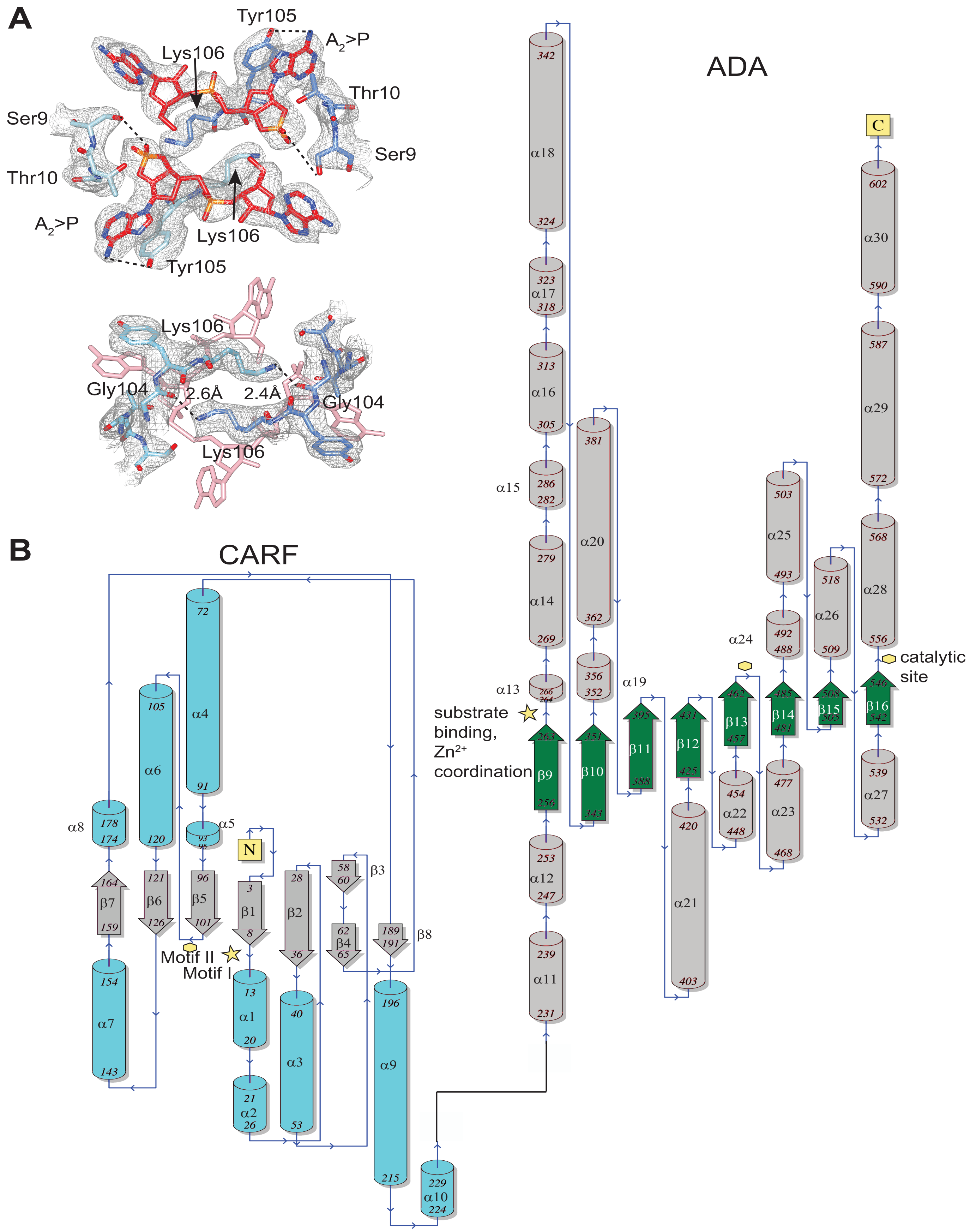

Appendix Figure S2. Structural properties of *Taq*Cad1-cA_4.

(A) Close-up view of the cA_4-bound site the dimer interface of the CARF domain. Top, density around both the bound A_2>P and the key binding residues. Bottom, close-up view highlighting the cross-subunit contacts of Lys106. **(B)** Topology of *Taq*Cad1 for both the CARF and the ADA domains. Key regions of interests are indicated.

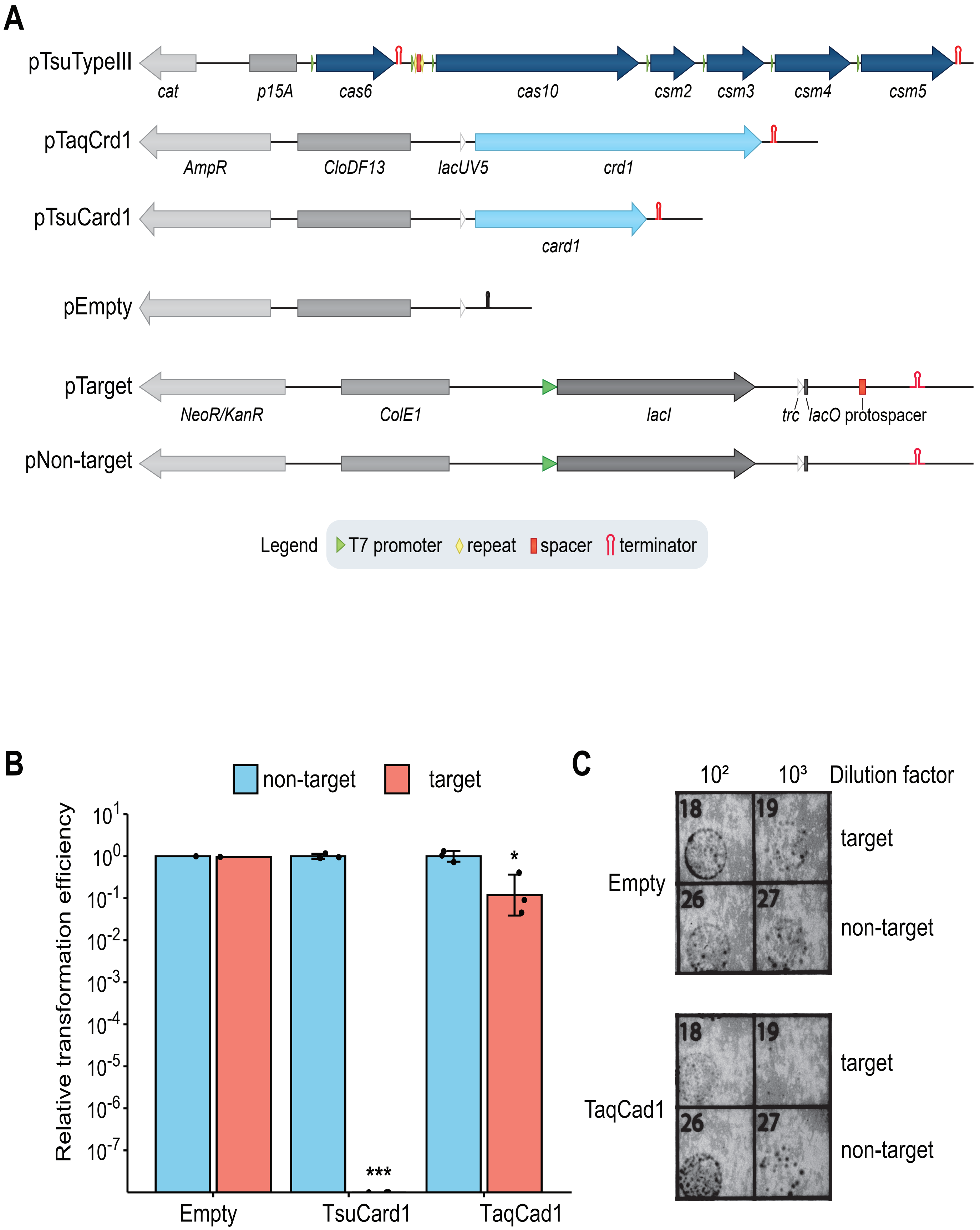

Appendix Figure S3. In vivo plasmid challenge assays to investigate the phenotype *Taq*Cad1 activation.

(A) Transformation efficiencies (relative to non-target control) of target and non-target plasmids in *E. coli* co-expressing *Taq*Cad1 and cA_4-producing Treponema succinifaciens type III CRISPR-Cas complex. Error bars represent the standard deviation of the mean. Statistical significance was calculated using Welch's t-test (n = 3 biological replicates). * p = 3.6E-2, *** p = 9.7E-6. **(B)** Comparison of colony sizes obtained from the assay after overnight growth. **(C)** Growth of transformants in liquid culture, measured as the optical density at 600 nm (OD600). Data represent the mean ± standard deviation of the mean (n = 3 biological replicates).

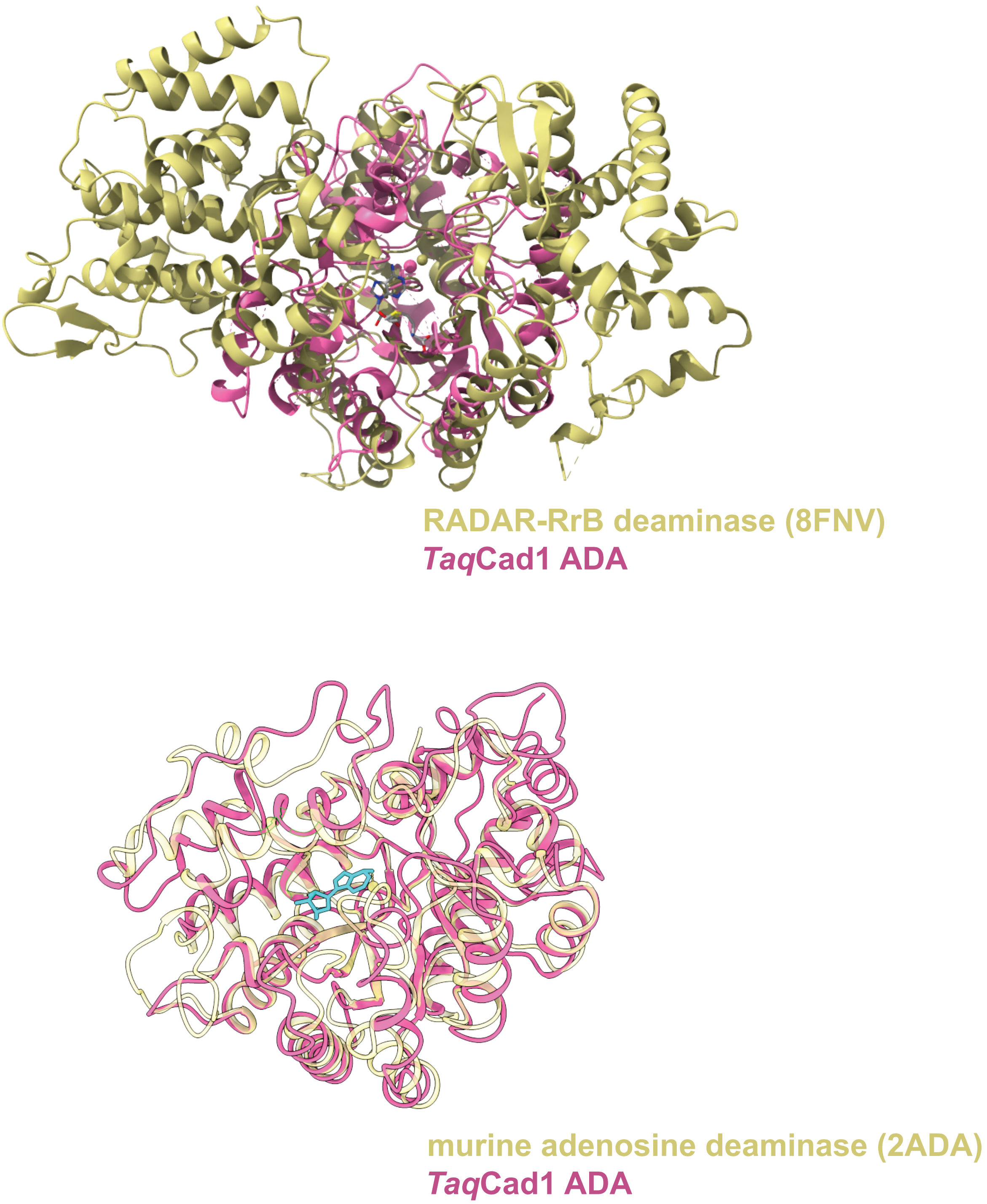

Appendix Figure S4. Structural comparison of *Taq*Cad1 with ADA.

Top, Comparison of the TaqCad1 ADA domain (pink) and the RrB subunit of the RADAR anti-phage system (yellow).
Bottom, Comparison of the TaqCad1 ADA domain (pink) and a murine adenosine deaminase (yellow). PDB codes are indicated.

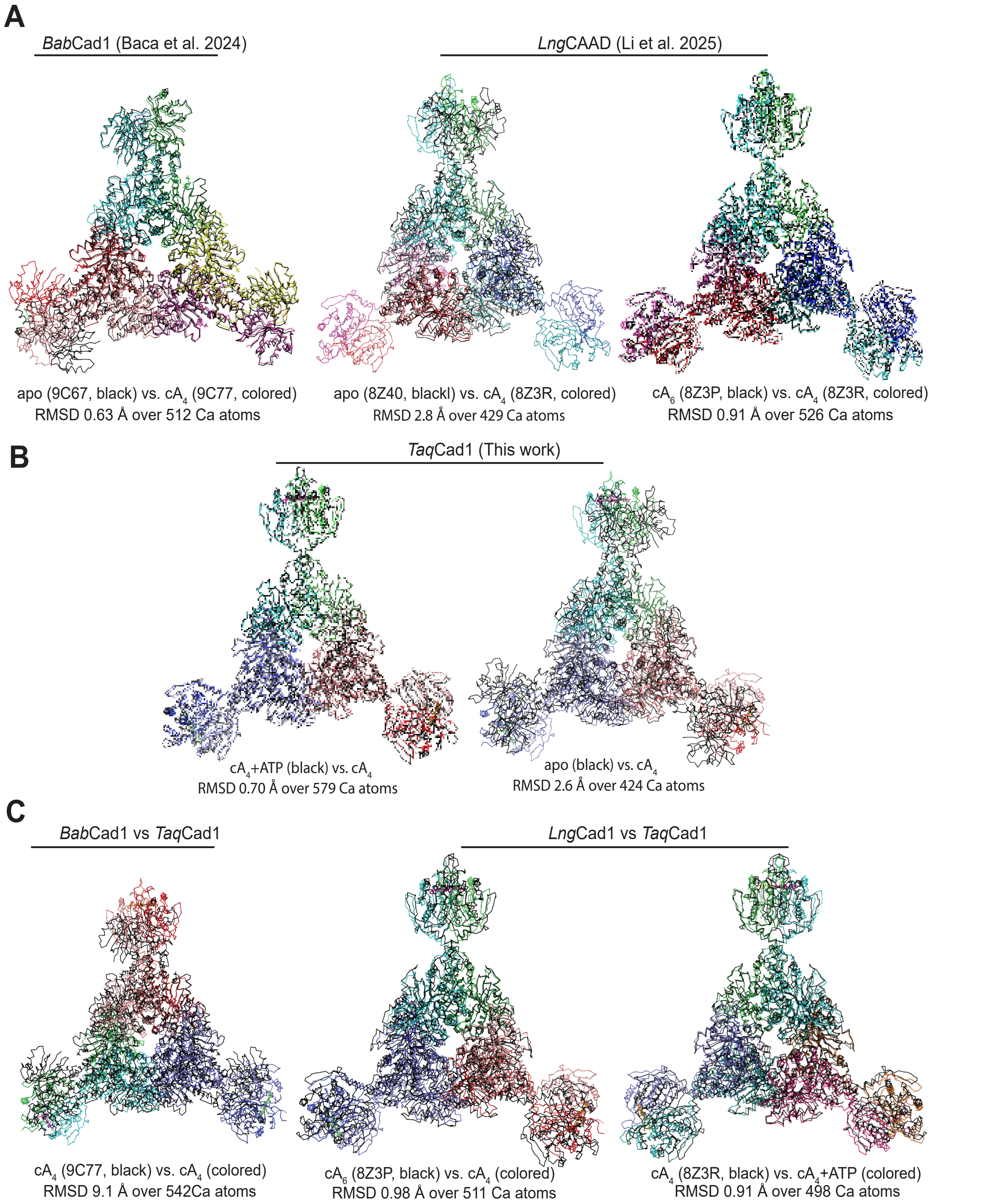

Appendix Figure S5. Structural comparison of *Taq*Cad1 with otherCad1 structures.

Root mean square (RMSD) and the aligned Cα atoms are listed under each pair of compared structures. PDB codes for the previously published Cad1 structures are listed. “This work” refers to the structures presented in this work. “cA_4” refers to the cA_4-bound structure. “cA_4+ATP” refers to the structure from the enzyme incubated with cA_4 and ATP. **(A)** Left, comparison of *Bab*Cad1 apo and cA_4-bound (cA_4) structures. Right, comparison of *Lng*CAAD structures. **(B)** Comparison of *Taq*Cad1 structures. **(C)** Comparison of *Taq*Cad1 structures to those of *Bab*Cad1 and *Lng*CAAD.

Appendix Table S1. Statistics of cryo-EM data collection and processing

Data collection & processing	$TaqCad1\text{-}cA_4$	ApoTaqCad1	$TaqCad1\text{-}cA_4\text{-}ATP$
Microscope	Titan Krios G3i	Titan Krios G3i	Titan Krios G3i
Detector	Gatan K3	Gatan K3	Gatan K3
Voltage	300 kV	300 kV	300 kV
Collecting mode	Counted super-resolution	Counted super-resolution	Counted super-resolution
Total dose (e^-/ $Å^2$)	60	60	50
Defocus range (μm)	(-0.8) - (-2.5)	(-0.8) - (-2.5)	(-1.0) - (-2.2)
Nominal magnification	81K	81K	105K
Frames collected per exposure	60	60	60
Frame-alignment software	MotionCor2-1.5.0	MotionCor2-1.5.0	MotionCor2-1.5.0
CTF estimation software	Gctf	Gctf	Gctf
Raw images collected	8597	4186	7862
2D classification software	cryoSPARC(v4.1.1)	cryoSPARC(v4.1.1)	cryoSPARC(v4.1.1)
3D classification software	Relion5.0	Relion5.0	Relion5.0
Final reconstruction software	cryoSPARC(v4.1.1)	cryoSPARC(v4.1.1)	cryoSPARC(v4.1.1)
Applied symmetry	C1	C1	C1
Resolution method	FSC 0.143 cutoff	FSC 0.143 cutoff	FSC 0.143 cutoff
Local resolution software	Cryosparc(v4.1.1) Local Resolution Estimation	Cryosparc(v4.1.1) Local Resolution Estimation	Cryosparc(v4.1.1) Local Resolution Estimation
Map and model visualization software	Pymol/Chimera/Chimera-X, Coot	Pymol/Chimera/Chimera-X, Coot	Pymol/Chimera/Chimera-X, Coot

Appendix Table S2. Statistics of model refinement

	TaqCad1-cOA$_4$ (sym expanded dimer composite)	TaqCad1-cOA$_4$ (good hexamer)	Apo TaqCad1 (sym expanded dimer, composite)	ApoTaqCad1 (good hexamer)	TaqCad1-cOA$_4$-ATP (sym expanded dimer, composite)	TaqCad1-cOA$_4$-ATP (good hexamer)	TaqCad1-cOA$_4$-ATP (one dimer)	TaqCad1-cOA$_4$-ATP (two dimer)
CC (mask)	0.86	0.83	0.79	0.81	0.77	0.76	0.83	0.83
RMSD (bond lengths/bond angles)	0.003/0.597	0.003/0.578	0.006/0.841	0.027/1.628	0.004/0.561	0.003/0.531	0.002/0.551	0.002/0.489
# of particles for final reconstruction	4,288,906	409,445	938,192	15,816	859,261	149,171	165,680	257,900
Final resolution (Å)	2.29-2.36	2.36	2.55-2.87	3.08	2.45-2.54	3.17	2.86	2.68
Ramachandran plot<br>Outliers<br>Allowed<br>Favored	 0.08<br>2.00<br>97.91	 0.08<br>3.46<br>96.46	 0.25<br>4.29<br>95.47	 0.52<br>4.45<br>95.03	 0.00<br>2.08<br>97.92	 0.17<br>4.35<br>95.48	 0.11<br>3.56<br>96.33	 0.19<br>3.54<br>96.27
Cβ Outliers (%)	0.0	0.0	0.0	0.61	0.0	0.0	0.0	0.0
ADP (B-factors)<br>Iso/Aniso (#)<br>Protein<br>Nucleotide<br>Ligand<br>Water<br>(min/max/mean)	 9542/0<br>0.0/89.7/32.3<br>0.0/22.5/12.7<br>56.5/57.7/57.1<br>---	 28755/0<br>0.0/213.9/80.5<br>0.0/29.6/11.6<br>62.6/72.2/66.6<br>---	 9530/0<br>6.6/154.4/62.9<br>---<br>77.7/89.1/83.4<br>---	 28590/0<br>76.6/499.6/201.0<br>---<br>169.6/188.0/178.2<br>---	 9662/0<br>27.0/202.7/102.2<br>22.2/66.3/47.4<br>88.7/126.2/107.5<br>43.6/80/4/68.39	 28970/0<br>36.4/327.0/161.1<br>32.8/96.7/65.5<br>119.4/198.2/162.2<br>67.6/187.3/126.7	 21982/0<br>3.9/238.3/90.8<br>25.7/66.6/43.6<br>71.2/176.5/104.3<br>30.1/93.6/66.1	 25543/0<br>0.0/258.8/59.7<br>20.9/60.0/38.4<br>35.7/139.0/73.4<br>12.2/49.6/31.5
MolProbity score	1.51	1.61	1.94	2.23	1.45	2.42	2.05	2.20
Clash score	9.12	6.75	13.01	25.0	7.89	41.9	20.38	28.94
Rotamer outlier (%)	0.00	0.00	0.00	0.00	0.00	0.00	0.00	0.00
FSC model (0/0.143/0.5)	--	2.4/2.5/2.6	--	3.1/3.1/3.4	--	3.0/3.1/3.5	2.80/2.80/3.1	2.6/2.7/2.9
Deposited EMDB codes	47886 (consensus)<br>47887 (CARF focused)<br>47888 (ADA focused)<br>47890 (composite)	48405	70419 (consensus)<br>70423 (CARF focused)<br>70424 (ADA focused)<br>70417 (composite)	48116	70425 (consensus)<br>70426 (CARF focused)<br>70427 (ADA focused)<br>70422 (composite)	70428	70429	70430
Deposited PDB codes	9EBT	9MMW	9OF1	9EKA	9OFB	9OFC	9OFD	9OFE

Appendix Table S3. Oligos Used in This Study

Mutation	Oligos	Source
D548A	Forward: GAACACCGCTAACCTGGGTATTAGCCAG<br>Reverse: CCAGGTTAGCGGTGTTCAGGGTAACTGC	Eurofins
N549D	Forward: GAACACCGATGACCTGGGTATTAGCCAG<br>Reverse: ACCCAGGTCATCGGTGTTCAGGGTAAC	Eurofins
K106A	Forward: GGCGGCTATGCAACCATTTCTGCAGCAATG<br>Reverse: GAAATGGTTGCATAGCCGCCTGCCAGAC	Eurofins
A411E	Forward: GCGCGTCATTTAGAGTTAGCAATTACCGCAG<br>Reverse: TTGCTAACTCTAAATGACGCGCAATACGAC	Eurofins
R408D	Forward: CGTAGTGATATTGCGCGTCATTTAGAGTTAG<br>Reverse: GCGCAATATCACTACGATCGCCGCCTTCTTC	Eurofins
G264D	Forward: GTCATTTAGACGGTTTTGCAACCCACGGCG<br>Reverse: CAAAACCGTCCTAAATGACAATGCAGTTCAAC	Eurofins
M311D	Forward: GAGCGCTATGATCGTCTGGGCGATAATAACGGTAG<br>Reverse: CCCAGACGATCATAGCGCTCTAAACCAATCGGTTC	Eurofins
G314F	Forward: TGCGTCTGTTCGATAATAACGGTAGCGCATTACTG<br>Reverse: GTTATTATCGAACAGACGCATATAGCGCTCTAAAC	Eurofins
*Taq*Csx1 cloning	Forward: actttaataaggagatatacATGGAAACGATGCCAAGGCAAG<br>Reverse: gcgcgccgagctcgaattcgTCACGCTTTGCCCTCCCC	Eurofins
RNA1 (RNA cleavage assay)	5’GGTTGGAAAGCCGGTTTTCTTTGATGTCACGGAACACGUUCUUUGAA CCAAGCUUCAAC 3’	IDT

Protein	Amino acid sequence
*Taq*Cad1 WT	MGHHHHHHGSSGMRILLCSVGTSWAVVPEAMQLLGSQGFDEVHVLTTASSKISPGVEQLLRYFEMHP<br>GPRFSISRVQDFEDLRSEQDHMLFEEVLWRWLLQRAPQAAHRYICLAGGYKTISAAMQRAAALFGAC<br>EVFHVLCEPRFGPQGNREASTLEEVEQAIATNALRFVRLGPEPGWPQLRLLSAPSFPLESTLQGPVH<br>WVRASDMRLRQHVEGVLERSRHILAAWEGISELPIPALAAWPPSHLRWLHEPLDPVQDKAWVQALPK<br>VELHCHLGGFATHGELLHKVRQEAANPESLPPVRAIPLPPGWPIPEEPIGLERYMRLGDNNGSALLK<br>DPGCLRAQCRLLYEALLADHVAYAEIRCSPANYASASRSPWVVLQEIRNHFQQAMEETPEDRRCHVN<br>LLLTATREEGGDRSRIARHLALAITAAEHWKNGCRVVGVDLAGFEDRTTRAAMFATDFEPVHRVGLA<br>VTVHAGENDDVEGIWQAVFKLSARRLGHALHLSRSPDLLRVVAERGIAVELCPYANLQIKGFPLDEE<br>QEGSETYPLRGYLAAGVAVTLNTDNLGISQASLTDNLLLTARLCPGITRLEVLKTQVFAAQAAFANQ<br>AERKALWARLAQVPVPTDTEQKNGNDAKASHQPR-
*Taq*Cad1 (N549D)	MGHHHHHHGSSGMRILLCSVGTSWAVVPEAMQLLGSQGFDEVHVLTTASSKISPGVEQLLRYFEMHP<br>GPRFSISRVQDFEDLRSEQDHMLFEEVLWRWLLQRAPQAAHRYICLAGGYKTISAAMQRAAALFGAC<br>EVFHVLCEPRFGPQGNREASTLEEVEQAIATNALRFVRLGPEPGWPQLRLLSAPSFPLESTLQGPVH<br>WVRASDMRLRQHVEGVLERSRHILAAWEGISELPIPALAAWPPSHLRWLHEPLDPVQDKAWVQALPK<br>VELHCHLGGFATHGELLHKVRQEAANPESLPPVRAIPLPPGWPIPEEPIGLERYMRLGDNNGSALLK<br>DPGCLRAQCRLLYEALLADHVAYAEIRCSPANYASASRSPWVVLQEIRNHFQQAMEETPEDRRCHVN<br>LLLTATREEGGDRSRIARHLALAITAAEHWKNGCRVVGVDLAGFEDRTTRAAMFATDFEPVHRVGLA<br>VTVHAGENDDVEGIWQAVFKLSARRLGHALHLSRSPDLLRVVAERGIAVELCPYANLQIKGFPLDEE<br>QEGSETYPLRGYLAAGVAVTLNTDDLGISQASLTDNLLLTARLCPGITRLEVLKTQVFAAQAAFANQ<br>AERKALWARLAQVPVPTDTEQKNGNDAKASHQPR
*Taq*Cad1 (D548A)	MGHHHHHHGSSGMRILLCSVGTSWAVVPEAMQLLGSQGFDEVHVLTTASSKISPGVEQLLRYFEMHP<br>GPRFSISRVQDFEDLRSEQDHMLFEEVLWRWLLQRAPQAAHRYICLAGGYKTISAAMQRAAALFGAC<br>EVFHVLCEPRFGPQGNREASTLEEVEQAIATNALRFVRLGPEPGWPQLRLLSAPSFPLESTLQGPVH<br>WVRASDMRLRQHVEGVLERSRHILAAWEGISELPIPALAAWPPSHLRWLHEPLDPVQDKAWVQALPK<br>VELHCHLGGFATHGELLHKVRQEAANPESLPPVRAIPLPPGWPIPEEPIGLERYMRLGDNNGSALLK<br>DPGCLRAQCRLLYEALLADHVAYAEIRCSPANYASASRSPWVVLQEIRNHFQQAMEETPEDRRCHVN<br>LLLTATREEGGDRSRIARHLALAITAAEHWKNGCRVVGVDLAGFEDRTTRAAMFATDFEPVHRVGLA<br>VTVHAGENDDVEGIWQAVFKLSARRLGHALHLSRSPDLLRVVAERGIAVELCPYANLQIKGFPLDEE<br>QEGSETYPLRGYLAAGVAVTLNTADLGISQASLTDNLLLTARLCPGITRLEVLKTQVFAAQAAFANQ<br>AERKALWARLAQVPVPTDTEQKNGNDAKASHQPR

*Taq*Cad1 (K106A)

```
MGHHHHHHGSSGMRILLCSVGTSWAVVPEAMQLLGSQGFDEVHVLTTASSKISPGVEQLLRYFEMHP
GPRFSISRVQDFEDLRSEQDHMLFEEVLWRWLLQRAPQAAHRYICLAGGYATISAAMQRAAALFGAC
EVFHVLCEPRFGPQGNREASTLEEVEQAIATNALRFVRLGPEPGWPQLRLLSAPSFPLESTLQGPVH
WVRASDMRLRQHVEGVLERSRHILAAWEGISELPIPALAAWPPSHLRWLHEPLDPVQDKAWVQALPK
VELHCHLGGFATHGELLHKVRQEAANPESLPPVRAIPLPPGWPIPEEPIGLERYMRLGDNNGSALLK
DPGCLRAQCRLLYEALLADHVAYAEIRCSPANYASASRSPWVVLQEIRNHFQQAMEETPEDRRCHVN
LLLTATREEGGDRSRIARHLALAITAAEHWKNGCRVVGVDLAGFEDRTTRAAMFATDFEPVHRVGLA
VTVHAGENDDVEGIWQAVFKLSARRLGHALHLSRSPDLLRVVAERGIAVELCPYANLQIKGFPLDEE
QEGSETYPLRGYLAAGVAVTLNTDNLGISQASLTDNLLLTARLCPGITRLEVLKTQVFAAQAAFANQ
AERKALWARLAQVPVPTDTEQKNGNDAKASHQPR
```

*Taq*Cad1 (R408D,A411E)

```
MGHHHHHHGSSGMRILLCSVGTSWAVVPEAMQLLGSQGFDEVHVLTTASSKISPGVEQLLRYFEMHP
GPRFSISRVQDFEDLRSEQDHMLFEEVLWRWLLQRAPQAAHRYICLAGGYKTISAAMQRAAALFGAC
EVFHVLCEPRFGPQGNREASTLEEVEQAIATNALRFVRLGPEPGWPQLRLLSAPSFPLESTLQGPVH
WVRASDMRLRQHVEGVLERSRHILAAWEGISELPIPALAAWPPSHLRWLHEPLDPVQDKAWVQALPK
VELHCHLGGFATHGELLHKVRQEAANPESLPPVRAIPLPPGWPIPEEPIGLERYMRLGDNNGSALLK
DPGCLRAQCRLLYEALLADHVAYAEIRCSPANYASASRSPWVVLQEIRNHFQQAMEETPEDRRCHVN
LLLTATREEGGDRSDIARHLELAITAAEHWKNGCRVVGVDLAGFEDRTTRAAMFATDFEPVHRVGLA
VTVHAGENDDVEGIWQAVFKLSARRLGHALHLSRSPDLLRVVAERGIAVELCPYANLQIKGFPLDEE
QEGSETYPLRGYLAAGVAVTLNTDNLGISQASLTDNLLLTARLCPGITRLEVLKTQVFAAQAAFANQ
AERKALWARLAQVPVPTDTEQKNGNDAKASHQPR
```

*Taq*Csx1

```
MPRQAINLGERGPHLLLVNLGLGQDKRGPLGYAETTYLMPDGSRYPTSLAGFALWKWLDATGRSPVA
VVFACTESAWNQKQEALRKHAAELGLEPSKISPPVILGLPQTLEDVWAMIEPMESRLRAYHDAAGPV
LHLDLTHAFRAIPLAHLLIALYFQERGLATVGVCGYGVFQEGQAETPYIDLSHLLHLARWAQAVRSF
RERFDTTGLAGLLEQYEREARHAAVQSGMIPPPEVRRLISAARLAGPYFAAGLPLELGVHVGQTLGQ
TTRESLEKAATKLVAAQQSLVLELFDALHPLTPGQVAKKSAKASLVLDKQELARQMRLLKLWLQAGL
PERALLVLREVVINRLLLAANPESWLEHGIREQAETVLNDVFRPEEPPLPSSPELKVLRSLWREITN
RRNPLAHAGMCKSEVDVGQLSEAVEELVEKLEALLELDEPWQELARQLSTSGEGKA-
```
