# Supplementary material for: The twist-and-squeeze activation of CARF-fused adenosine deaminase by cyclic oligoadenylates
Source: EMBO J. 2025 Oct 17;44(23):6919–43. doi: 10.1038/s44318-025-00578-y (PMC12669630; doi:10.1038/s44318-025-00578-y)
Supplement: Supplementary file 5 — Movie EV3 [file 44318_2025_578_MOESM5_ESM.zip › Movie EV3 Legend.docx]

**Movie EV3**. **Related to Figures 4, 5, 6, & 7.** A major principal component motion of the cA_4_-, ATP-bound *Taq*Cad1 dimer from 3D variability analysis.
