# Supplementary material for: The twist-and-squeeze activation of CARF-fused adenosine deaminase by cyclic oligoadenylates
Source: EMBO J. 2025 Oct 17;44(23):6919–43. doi: 10.1038/s44318-025-00578-y (PMC12669630; doi:10.1038/s44318-025-00578-y)
Supplement: Supplementary file 6 — Source data Fig. 1 [file 44318_2025_578_MOESM6_ESM.zip › Figure 1/1B/rna cleavage assay csx1-varying Cad1.pdf]

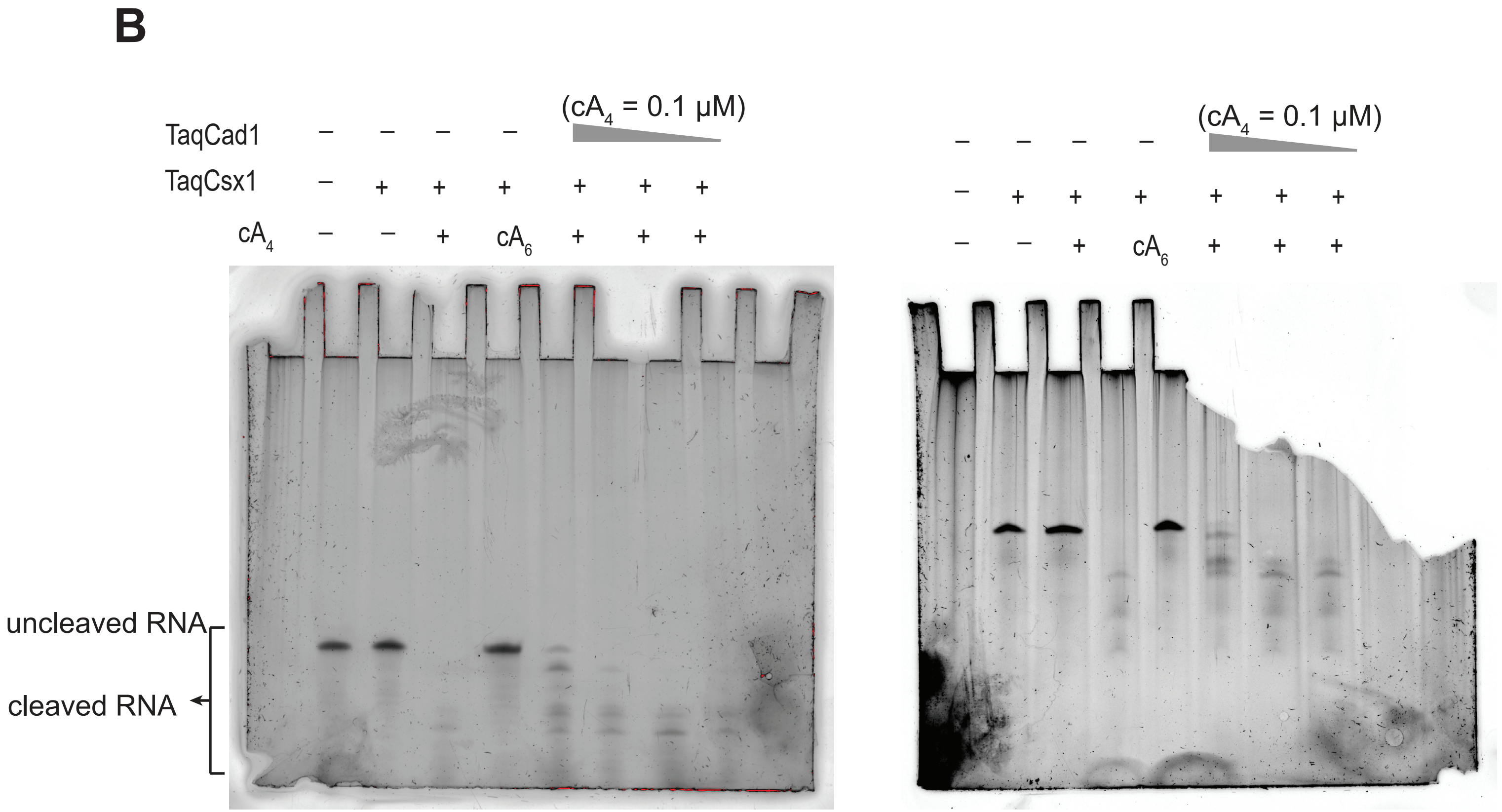

Figure Source Data Figure 1. Related to Figures 1d. Activation of *Taq*Csx1 by cA₄ and the impact of *Taq*Cad1 ring nuclease activity on *Taq*Csx1 RNA cleavage activity.

(B) Replicates showing activation of *Taq*Csx1 by cA_4 and the impact of *Taq*Cad1 ring nuclease activity on *Taq*Csx1 RNA cleavage activity at varying concentrations of *Taq*Cad1 using 0.1 μM of cA_4.
