# Supplementary material for: The twist-and-squeeze activation of CARF-fused adenosine deaminase by cyclic oligoadenylates
Source: EMBO J. 2025 Oct 17;44(23):6919–43. doi: 10.1038/s44318-025-00578-y (PMC12669630; doi:10.1038/s44318-025-00578-y)
Supplement: Supplementary file 7 — Source data Fig. 2 [file 44318_2025_578_MOESM7_ESM.zip › Figure 2/2A/uv absorbance-adenosine derivatives.pdf]

A

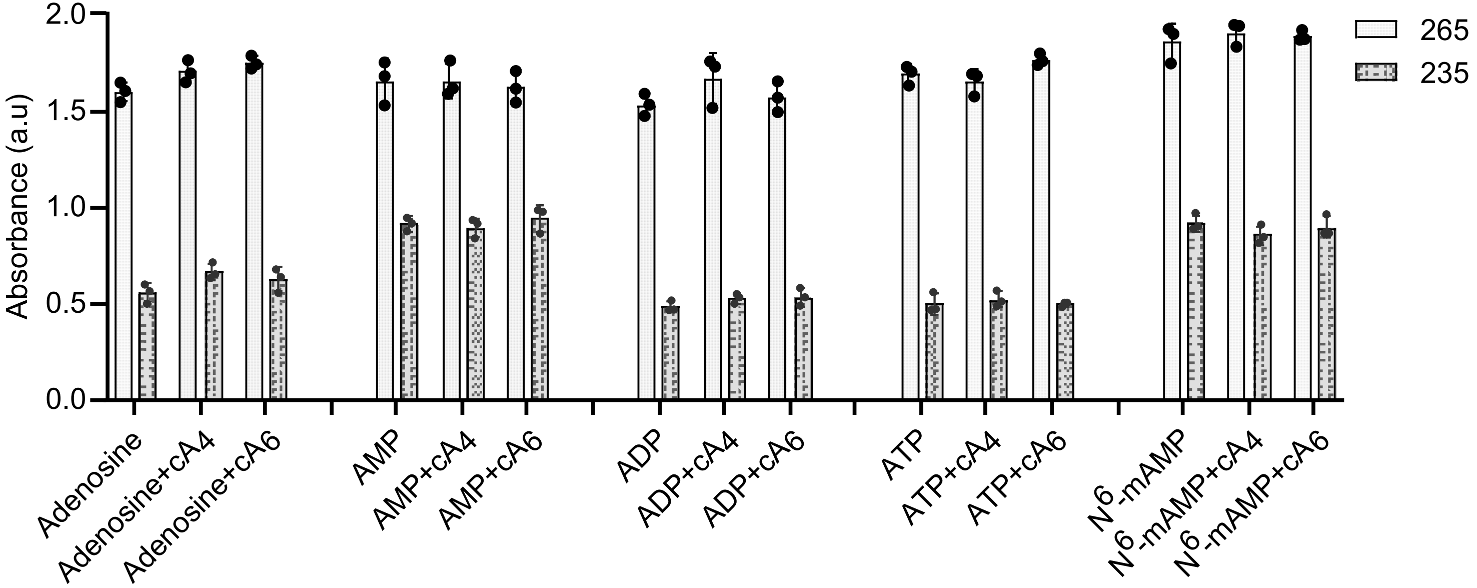

Figure Source Data Figure 2. Related to Figures 2. UV absorbance analysis of *Taq*Cad1 deamination activities.

UV absorbance at wavelength 265 and 235 nm measures the characteristic absorbance of adenosine and inosine, respectively. "WT+ATP" indicates the reaction in the absence of any ligand. *Taq*Cad1(WT)-mediated reactions were dephosphorylated by calf intestinal alkaline phosphatase following heat deactivation **(A)** Detection of spectroscopic changes by using cA_4 and cA_6 with Adenosine, AMP, ADP, N^6-methyl-AMP (N^6-mAMP) and ATP.
