# Supplementary material for: The twist-and-squeeze activation of CARF-fused adenosine deaminase by cyclic oligoadenylates
Source: EMBO J. 2025 Oct 17;44(23):6919–43. doi: 10.1038/s44318-025-00578-y (PMC12669630; doi:10.1038/s44318-025-00578-y)
Supplement: Supplementary file 8 — Source data Fig. 3 [file 44318_2025_578_MOESM8_ESM.zip › Figure 3/mass spec- cad1 ring nuclease products.pdf]

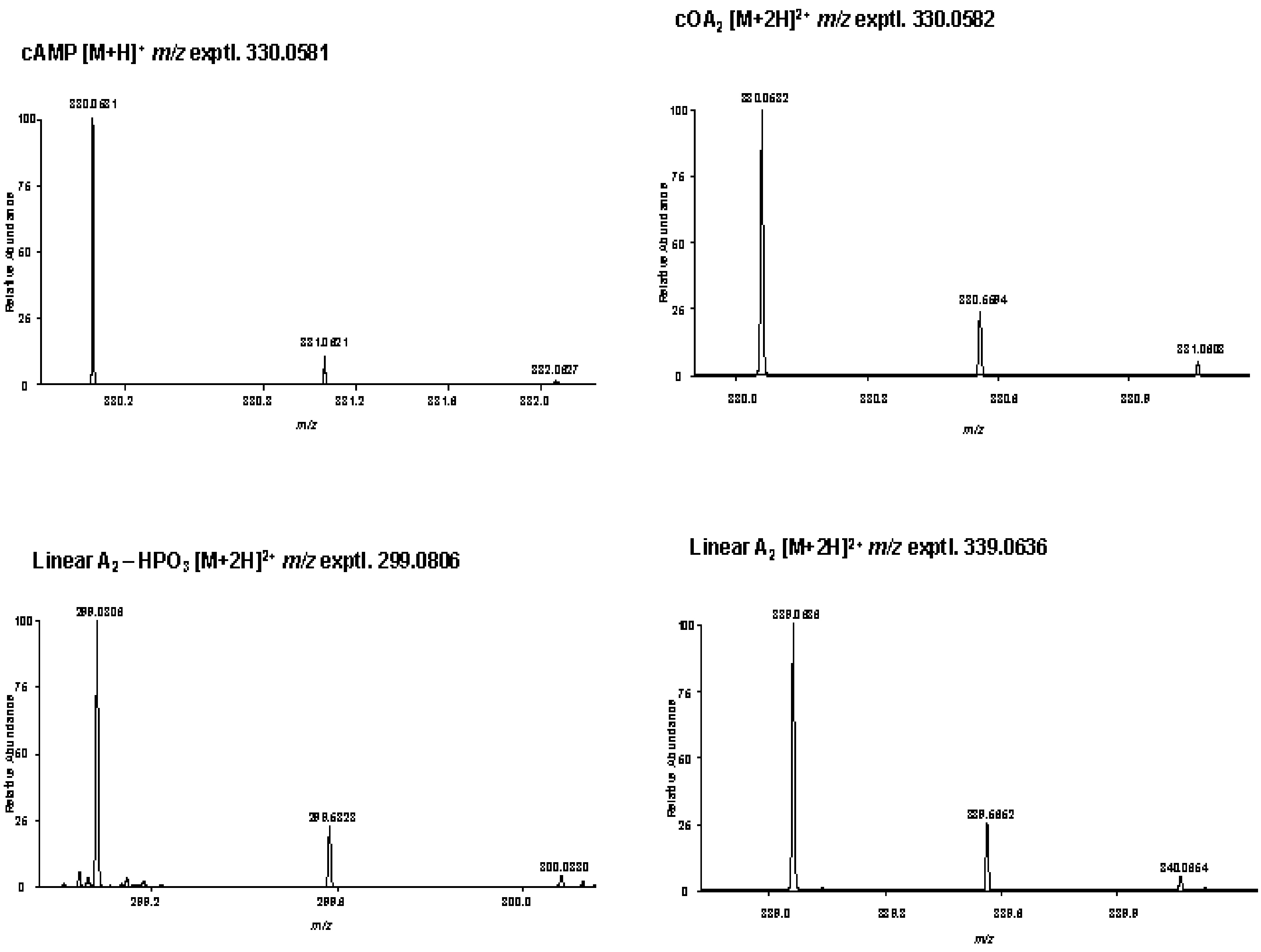

Figure Source Data Figure 3. Related to Appendix Figure S1D.

Mass spectrometry anaylsis of ring nuclease products of *Taq*Cad1 upon incubating with cA$_4$ showing the mass to charge ratios and relative abundances of each detected product.
