# Supplementary material for: The twist-and-squeeze activation of CARF-fused adenosine deaminase by cyclic oligoadenylates
Source: EMBO J. 2025 Oct 17;44(23):6919–43. doi: 10.1038/s44318-025-00578-y (PMC12669630; doi:10.1038/s44318-025-00578-y)
Supplement: Supplementary file 10 — Expanded View Figures [file 44318_2025_578_MOESM10_ESM.pdf]

Expanded View Figures

Figure EV1. Concentration effects by cA_4 and $A_2>P$ in their activation of *Taq*Cad1.

"WT + ATP" indicates the reaction in the absence of any ligand. *Taq*Cad1-mediated reactions were dephosphorylated by calf intestinal alkaline phosphatase following heat deactivation. (**A**) UV absorption measurement results of the reaction of *Taq*Cad1 with ATP in the presence of various of cA_4 concentrations (0.2, 0.5, and 2 µM). (**B**) UV absorption measurement results of the reaction of *Taq*Cad1 with ATP in the presence of various of products of 20 µM cA_4 incubated with the K106A variant for 15, 30, and 60 min, respectively. (**C**) UV absorption measurement results of the reaction of *Taq*Cad1 with ATP in the presence of ring nuclease products resulted from incubating cA_4 at 5, 10, and 20 µM, respectively, with 2 µM WT *Taq*Cad1 for 30 (top) and 60 (bottom) min, respectively. For panels (**A–C**), data represent the mean ± standard deviation of the mean ($n=3$ biological replicates). Source data are available online for this figure.

© The Author(s)

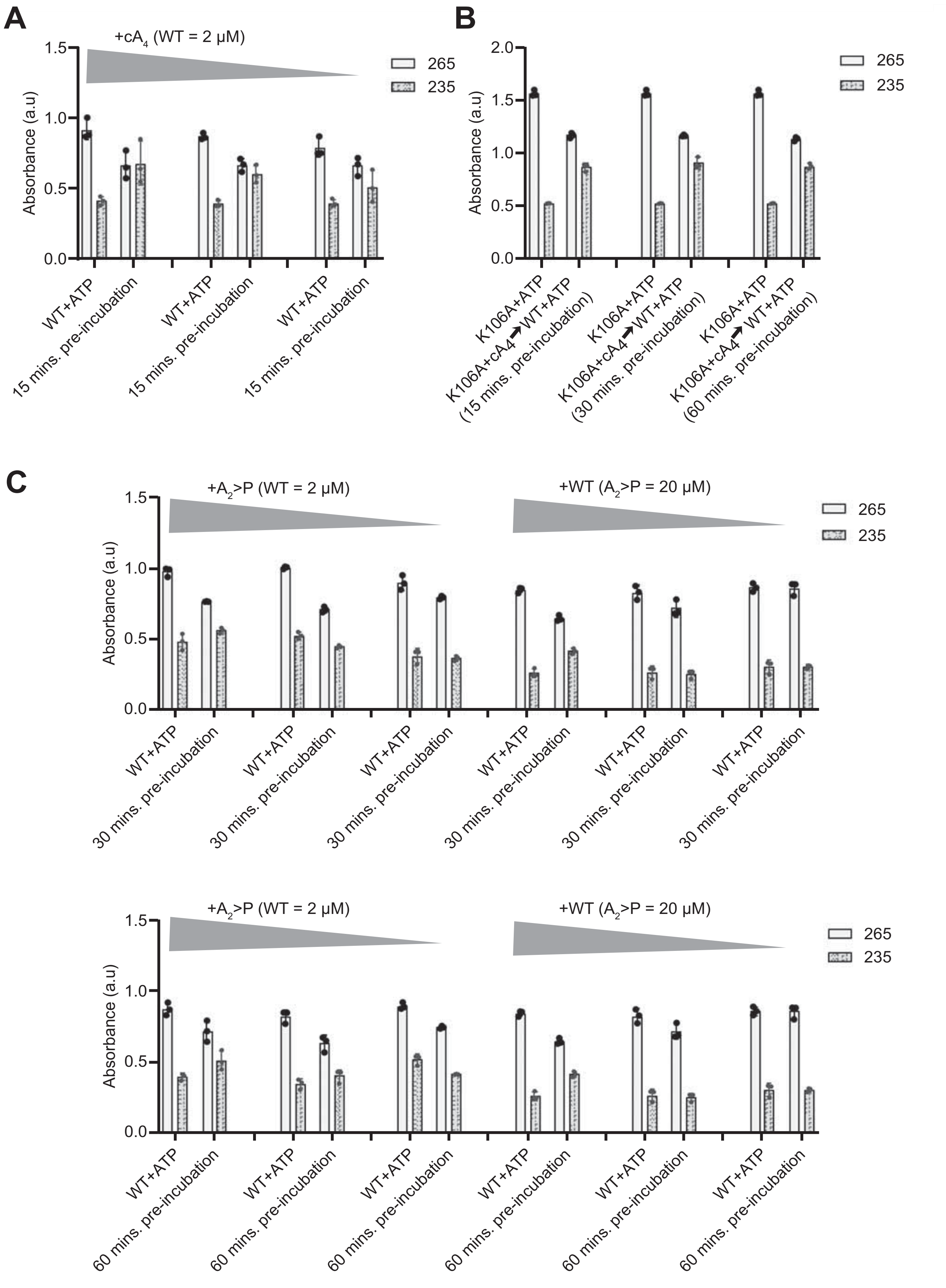
A
+cA4 (WT = 2 μM)
Absorbance (a.u)
265
235
WT+ATP
15 mins. pre-incubation
B
265
235
K106A+ATP
K106A+cA4 ➚ WT+ATP
(15 mins. pre-incubation)
(30 mins. pre-incubation)
(60 mins. pre-incubation)
C
+A2>P (WT = 2 μM)
+WT (A2>P = 20 μM)
30 mins. pre-incubation
60 mins. pre-incubation

 © The Author(s)

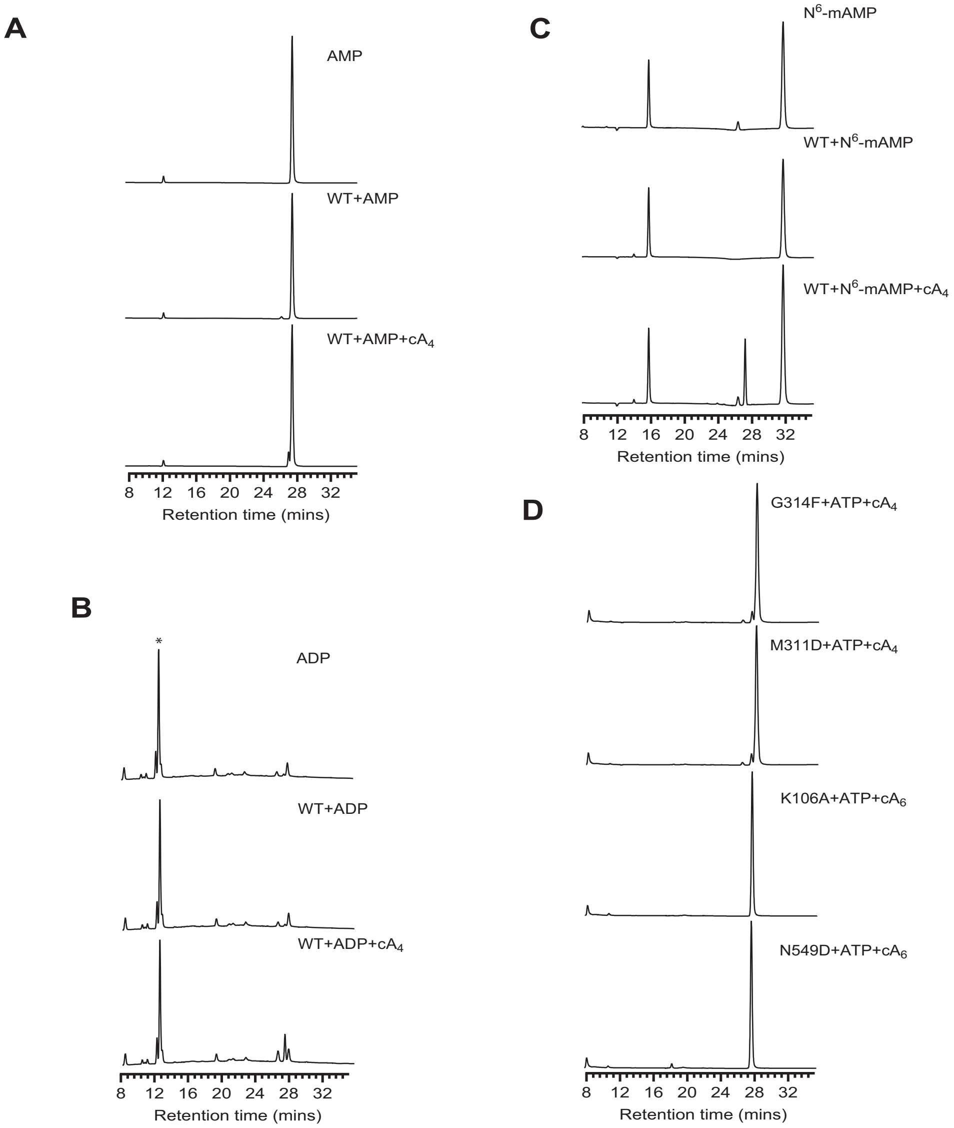

Figure EV2. Additional deamination assays for *Taq*Cad1.

(**A**) HPLC profiles following *Taq*Cad1 reaction with adenosine monophosphate (AMP) in the presence and absence of cA_4. (**B**) HPLC profiles following *Taq*Cad1 reaction with adenosine diphosphate (ADP) in the presence and absence of cA_4. The asterisk indicates a nonspecific peak present in the unreacted ADP sample (top). (**C**) HPLC profiles following *Taq*Cad1 reaction with N^6-methyl-adenosine monophosphate (N^6m-AMP) in the presence and absence of cA_4. (**D**) HPLC profiles of the reaction products following incubating ATP with *Taq*Cad1 variants (G314F, M311D, N549D, or K106A) in the presence and absence of cA_4 or cA_6. The reaction products were treated with CIP. The profiles of adenosine and inosine are included for comparison. Dash lines indicate the elution positions of inosine and adenosine.

© The Author(s)

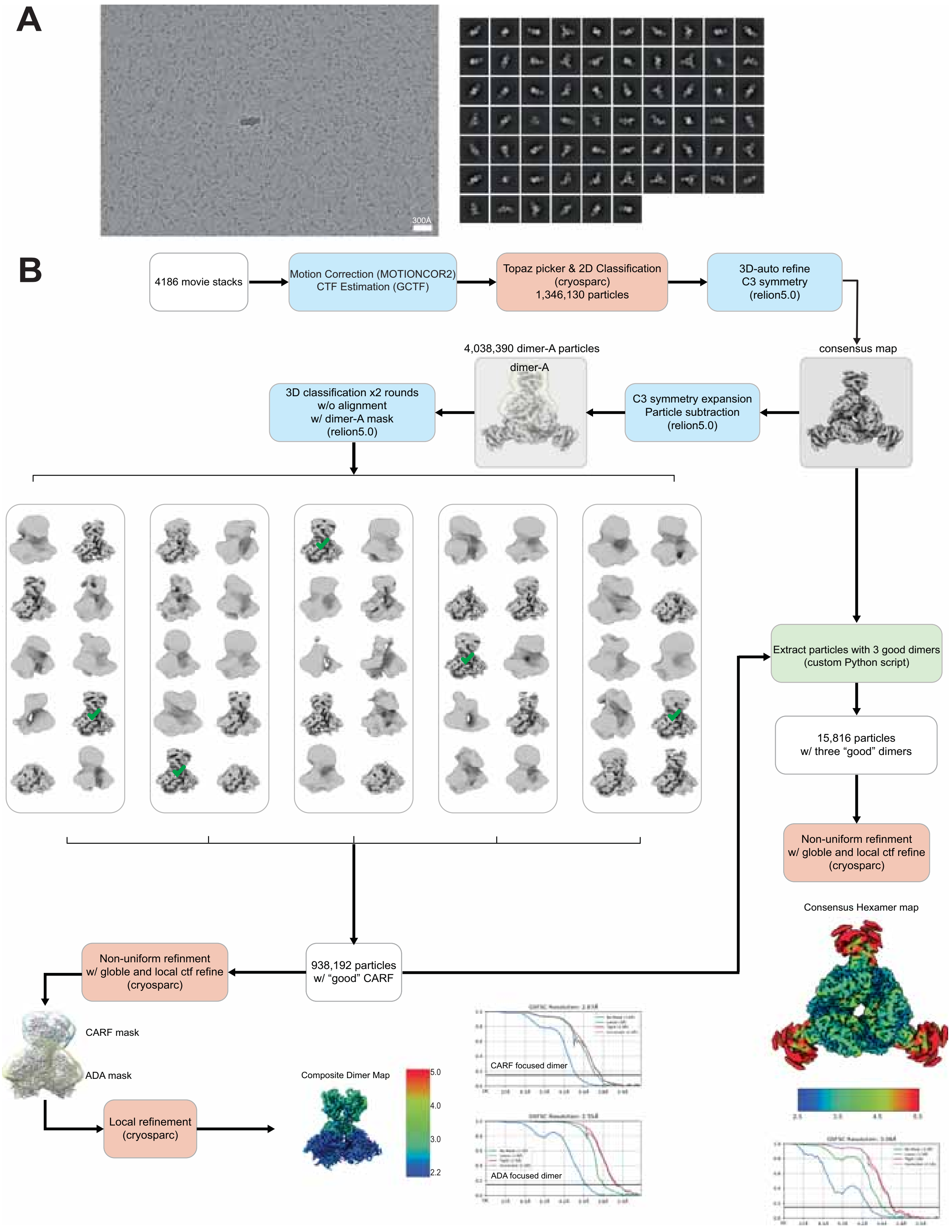
A
300Å
B
4186 movie stacks
Motion Correction (MOTIONCOR2)
CTF Estimation (GCTF)
Topaz picker & 2D Classification
(cryosparc)
1,346,130 particles
3D-auto refine
C3 symmetry
(relion5.0)
consensus map
C3 symmetry expansion
Particle subtraction
(relion5.0)
4,038,390 dimer-A particles
dimer-A
3D classification x2 rounds
w/o alignment
w/ dimer-A mask
(relion5.0)
Extract particles with 3 good dimers
(custom Python script)
15,816 particles
w/ three "good" dimers
Non-uniform refinment
w/ globle and local ctf refine
(cryosparc)
Consensus Hexamer map
938,192 particles
w/ "good" CARF
Non-uniform refinment
w/ globle and local ctf refine
(cryosparc)
CARF mask
ADA mask
Local refinement
(cryosparc)
Composite Dimer Map
5.0
4.0
3.0
2.2
CARF focused dimer
ADA focused dimer

Figure EV3. Data collection, processing, and 3D reconstruction of the cryoEM structures of apo *Taq*Cad1.

(**A**) Example micrograph and 2D class averages (scale bar 300 Å). (**B**) Data collection, particle selection, and reconstruction flowchart. Two reconstructions were made for the individual dimers and the intact hexamer, respectively. The dimer was reconstructed from a C3 symmetry expansion, leading to over 4 million particles. 3D classification was performed to select the best dimers that were then reconstructed for the CARF and ADA domains, respectively, that were combined to form the composite map. The best hexamer containing three intact dimers was reconstructed without focused refinement. Final maps used for refinement are shown with local resolutions and their Fourier shell correlation (FSC) curves. 0.143 FSC cutoff was used for resolution estimation.

© The Author(s)

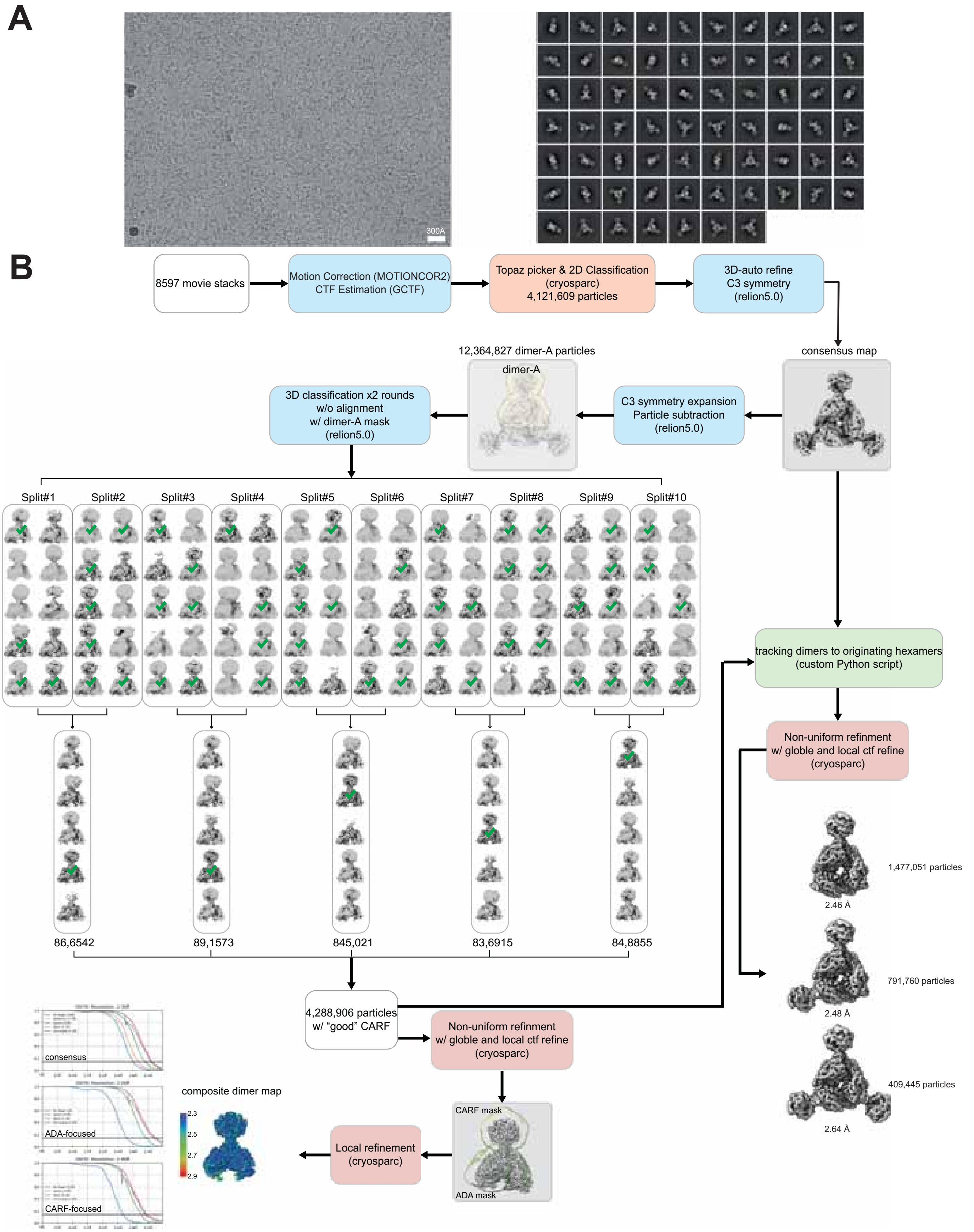
A
300A
B
8597 movie stacks
Motion Correction (MOTIONCOR2)
CTF Estimation (GCTF)
Topaz picker & 2D Classification
(cryosparc)
4,121,609 particles
3D-auto refine
C3 symmetry
(relion5.0)
consensus map
12,364,827 dimer-A particles
dimer-A
C3 symmetry expansion
Particle subtraction
(relion5.0)
3D classification x2 rounds
w/o alignment
w/ dimer-A mask
(relion5.0)
Split#1
Split#2
Split#3
Split#4
Split#5
Split#6
Split#7
Split#8
Split#9
Split#10
86,6542
89,1573
845,021
83,6915
84,8855
4,288,906 particles
w/ "good" CARF
Non-uniform refinment
w/ globle and local ctf refine
(cryosparc)
CARF mask
ADA mask
Local refinement
(cryosparc)
composite dimer map
2.3
2.5
2.7
2.9
consensus
ADA-focused
CARF-focused
tracking dimers to originating hexamers
(custom Python script)
Non-uniform refinment
w/ globle and local ctf refine
(cryosparc)
1,477,051 particles
2.46 Å
791,760 particles
2.48 Å
409,445 particles
2.64 Å

© The Author(s)

Figure EV4. Data collection, processing, and 3D reconstruction of the cryoEM structures of *Taq*Cad1 incubated with cA_4.

(**A**) Example micrograph and 2D class averages (scale bar 300 Å). (**B**) Data collection, particle selection, and reconstruction flowchart. Two reconstructions were made for the individual dimers and the intact hexamer, respectively. The dimer was reconstructed from a C3 symmetry expansion, leading to over 4 million particles. 3D classification was performed to select the best dimers that were then reconstructed for the CARF and ADA domains, respectively, that were combined to form the composite map. The hexamers containing one, two, or three intact dimers were reconstructed without focused refinement. Final maps used for refinement are shown with local resolutions and their Fourier shell correlation (FSC) curves. 0.143 FSC cutoff was used for resolution estimation.

© The Author(s)

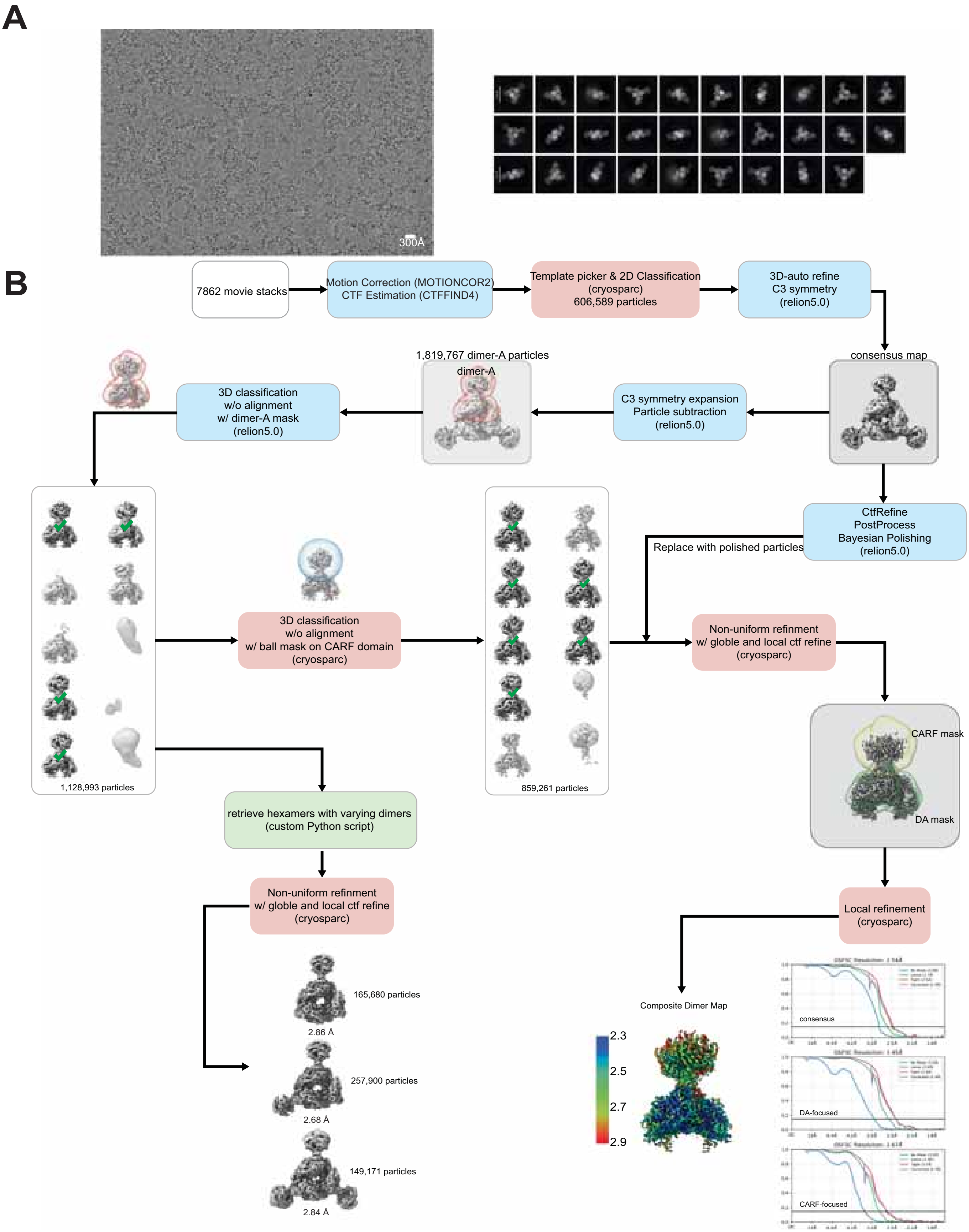
A
300A
B
7862 movie stacks
Motion Correction (MOTIONCOR2)
CTF Estimation (CTFFIND4)
Template picker & 2D Classification
(cryosparc)
606,589 particles
3D-auto refine
C3 symmetry
(relion5.0)
consensus map
C3 symmetry expansion
Particle subtraction
(relion5.0)
1,819,767 dimer-A particles
dimer-A
3D classification
w/o alignment
w/ dimer-A mask
(relion5.0)
CtfRefine
PostProcess
Bayesian Polishing
(relion5.0)
Replace with polished particles
1,128,993 particles
3D classification
w/o alignment
w/ ball mask on CARF domain
(cryosparc)
859,261 particles
Non-uniform refinment
w/ globle and local ctf refine
(cryosparc)
CARF mask
DA mask
retrieve hexamers with varying dimers
(custom Python script)
Non-uniform refinment
w/ globle and local ctf refine
(cryosparc)
Local refinement
(cryosparc)
165,680 particles
2.86 Å
257,900 particles
2.68 Å
149,171 particles
2.84 Å
Composite Dimer Map
2.3
2.5
2.7
2.9
consensus
DA-focused
CARF-focused

 © The Author(s)

Figure EV5. Data collection, processing, and 3D reconstruction of the cryoEM structures of *Taq*Cad1 incubated with cA_4 and ATP.

(**A**) Example micrograph and 2D class averages (scale bar 300 Å). (**B**) Data collection, particle selection, and reconstruction flowchart. Two reconstructions were made for the individual dimers and the intact hexamer, respectively. The dimer was reconstructed from a C3 symmetry expansion, leading to over 4 million particles. 3D classification was performed to select the best dimers that were then reconstructed for the CARF and ADA domains, respectively, that were combined to form the composite map. The hexamers containing one, two, or three intact dimers were reconstructed without focused refinement. Final maps used for refinement are shown with local resolutions and their Fourier shell correlation (FSC) curves. 0.143 FSC cutoff was used for resolution estimation.

© The Author(s)
